# Supplementary material for: The transcriptional regulator Ume6 is a major driver of early gene expression during gametogenesis
Source: Genetics. 2023 Jul 11;225(2):iyad123. doi: 10.1093/genetics/iyad123 (PMC10550318; doi:10.1093/genetics/iyad123)
Supplement: iyad123_Supplementary_Data [file iyad123_supplementary_data.zip › Tables_S4-S6_GENETICS-2023-306081.docx]

**Table S4. Strains used in this study.**

| Strain | *Genotype* |
| --- | --- |
| UB13 | *ho::LYS2 lys2 ura3 leu2::hisG his3::hisG trp1::hisG* ***(SK1 wild-type)*** |
| UB95 | *MAT***a***/MATalpha  ndt80::pGAL-NDT80::TRP1/ndt80::pGAL-NDT80::TRP1;  ura3::pGPD1-GAL4(848).ER::URA3/ura3::pGPD1-GAL4(848).ER::URA3* |
| UB3301 | *MAT***a***; MATalpha;  ime1::pCUP-IME1::HphMX/ime1::pCUP-IME1::HphMX;  ime4::pCUP-IME4::NAT/ime4::pCUP-IME4::NAT;  ume6::UME6-3V5::His3MX/ume6::UME6-3V5::His3MX* |
| UB17716 | *MAT***a***; trp1::TRP1; his3::HIS3* |
| UB18216 | *MAT***a***;  ume6::UME6-IAA7-3V5::KanMX; trp1::TRP1; his3::HIS3* |
| UB18287 | *MATa;  ume6::UME6-IAA7-3V5::KanMX;  trp1::pGPD1-LexA-ER-HA-B112::TRP1;  his3::HIS3* |
| UB17646 | *MAT***a***;  ume6::UME6-IAA7-3V5::KanMX; trp1::pGPD1-LexA-ER-HA-B112::TRP1;  his3::4xplexO-OsTIR1(ScOP)::HIS3* |
| UB17718 | *MAT***a***;  ume6::hygBMX ; trp1::TRP1; his3::HIS3* |
| UB19103 | *MAT***a***/alpha;  ime1::pCUP-IME1::NATMX/ime1::pCUP-IME1::NATMX;  ime4::pCUP-IME4::NATMX/ime4::pCUP-IME4::NATMX;  trp1::TRP1/trp1::TRP1;  his3::HIS3/his3::HIS3* |
| UB19101 | *MAT***a***/alpha;  ime1::pCUP-IME1::NATMX/ime1::pCUP-IME1::NATMX;  ime4::pCUP-IME4::NATMX/ime4::pCUP-IME4::NATMX;  ume6::Ume6-AID-3v5::KanMX/ume6::Ume6-AID-3v5::KanMX; trp1::TRP1/trp1::TRP1; his3::HIS3/his3::HIS3* |
| UB19105 | *MAT***a***/alpha;  ime1::HygBMX/ime1::HygBMX;  trp1::TRP1/trp1::TRP1; his3::HIS3/his3::HIS3* |
| UB25688 | *MAT***a***/alpha;  ime1::pCUP-IME1::NATMX/ime1::pCUP-IME1::NATMX;  ime4::pCUP-IME4::NATMX/ime4::pCUP-IME4::NATMX;  ume6::UME6-IAA7-3V5::KanMX/ume6::UME6-IAA7-3V5::KanMX;  trp1::pGPD1-LexA-ER-HA-GAL4(770-881)::TRP1/trp1::pGPD1-LexA-ER-HA-GAL4(770-881)::TRP1; his3::HIS3/his3::HIS3* |
| UB25092 | *MAT***a***/alpha;  ime1::pCUP-IME1::NATMX/ime1::pCUP-IME1::NATMX;  ime4::pCUP-IME4::NATMX/ime4::pCUP-IME4::NATMX;  ume6::UME6-IAA7-3V5::KanMX/ume6::UME6-IAA7-3V5::KanMX;  trp1::pGPD1-LexA-ER-HA-GAL4(770-881)::TRP1/trp1::pGPD1-LexA-ER-HA-GAL4(770-881)::TRP1; his3::8xplexO-OSTIR1(ScOP)::HIS3/his3::8xplexO-OSTIR1(ScOP)::HIS3* |
| UB26621 | *MAT***a***/alpha; ura3::URA3/ura3::URA3* |
| UB26637 | *MAT***a***/alpha;  ime1::sfGFP-IME1/ime1::sfGFP-IME1;  ura3::URA3/ura3::URA3* |
| UB26625 | *MAT***a***/alpha;  ume6::UME6-3v5::KanMX/ume6::UME6-3v5::KanMX;  ura3::URA3/ura3::URA3* |
| UB26641 | *MAT***a***/alpha;  ime1::sfGFP-IME1/ime1::sfGFP-IME1;  ume6::UME6-3v5::KanMX/ume6::UME6-3v5::KanMX;  ura3::URA3/ura3::URA3* |
| UB26629 | *MAT****a****/alpha;  ume6::UME6(T99N)-3v5::KanMX/ume6::UME6(T99N)-3v5::KanMX;  ura3::URA3/ura3::URA3* |
| UB26645 | *MAT****a****/alpha;  ime1::sfGFP-IME1/ime1::sfGFP-IME1;  ume6::UME6(T99N)-3v5::KanMX/ume6::UME6(T99N)-3v5::KanMX;  ura3::URA3/ura3::URA3* |
| UB27313 | *MAT***a***/alpha;  ume6::UME6(T99N)-antiGFP(VH16)::URA3/ume6::UME6(T99N)-antiGFP(VH16)::URA3* |
| UB27243 | *MAT***a***/alpha;  ime1::sfGFP-IME1/ime1::sfGFP-IME1;  ume6::UME6(T99N)-antiGFP(VH16)::URA3/ume6::UME6(T99N)-antiGFP(VH16)::URA3* |
| UB32574 | *MAT***a***/alpha;  ime1::HygBMX/ime1::HygBMX;  his3::pIME1-IME1::HIS3/his3::pIME1-IME1::HIS3;  ume6::UME6(T99N)-antiGFP(VH16)::URA3/ume6::UME6(T99N)-antiGFP(VH16)::URA3* |
| UB32572 | *MAT***a***/alpha;  ime1::HygBMX/ime1::HygBMX;  his3::pIME1-sfGFP-IME1::HIS3/his3::pIME1-sfGFP-IME1::HIS3;  ume6::UME6(T99N)-antiGFP(VH16)::URA3/ume6::UME6(T99N)-antiGFP(VH16)::URA3* |
| UB33044 | *MAT***a***/alpha;  ime1::HygBMX/ime1::HygBMX;  his3::pIME1-SV40-GAL4(75-881)::HIS3/his3::pIME1-SV40-GAL4(75-881)::HIS3;  ume6::UME6(T99N)-antiGFP(VH16)::URA3/ume6::UME6(T99N)-antiGFP(VH16)::URA3* |
| UB30293 | *MAT***a***/alpha;  ime1::HygBMX/ime1::HygBMX;  his3::pIME1-sfGFP-SV40-GAL4(75-881)::HIS3/his3::pIME1-sfGFP-SV40-GAL4(75-881)::HIS3;  ume6::UME6(T99N)-antiGFP(VH16)::URA3/ume6::UME6(T99N)-antiGFP(VH16)::URA3* |
| UB33048 | *MAT***a***/alpha;  ime1::HygBMX/ime1::HygBMX;  his3::pIME1-SV40-B112::HIS3/his3::pIME1-SV40-B112::HIS3;  ume6::UME6(T99N)-antiGFP(VH16)::URA3/ume6::UME6(T99N)-antiGFP(VH16)::URA3* |
| UB30295 | *MAT***a***/alpha;  ime1::HygBMX/ime1::HygBMX;  his3::pIME1-sfGFP-SV40-B112::HIS3/his3::pIME1-sfGFP-SV40-B112::HIS3;  ume6::UME6(T99N)-antiGFP(VH16)::URA3/ume6::UME6(T99N)-antiGFP(VH16)::URA3* |
| UB31727 | *MAT***a***/alpha;  ime1::HygBMX/ime1::HygBMX;  his3::pIME1-sfGFP-SV40-GAL4(75-881)::HIS3/his3::pIME1-sfGFP-SV40-GAL4(75-881)::HIS3;  ume6::UME6(T99N)-antiGFP(VH16)::URA3/ume6::UME6(T99N)-antiGFP(VH16)::URA3;  htb1::HTB1-mCherry::HISMX6/htb1::HTB1-mCherry::HISMX6* |
| UB31729 | *MAT***a***/alpha;  ime1::HygBMX/ime1::HygBMX;  his3::pIME1-sfGFP-SV40-B112::HIS3/his3::pIME1-sfGFP-SV40-B112::HIS3;  ume6::UME6(T99N)-antiGFP(VH16)::URA3/ume6::UME6(T99N)-antiGFP(VH16)::URA3; htb1::HTB1-mCherry::HISMX6/htb1::HTB1-mCherry::HISMX6* |
| UB33625 | *MAT***a***/alpha;  ime1::HygBMX/ime1::HygBMX;  his3::pIME1-sfGFP-IME1::HIS3/his3::pIME1-sfGFP-IME1::HIS3;  ume6::UME6(T99N)-antiGFP(VH16)::URA3/ume6::UME6(T99N)-antiGFP(VH16)::URA3; htb1::HTB1-mCherry::HISMX6/htb1::HTB1-mCherry::HISMX6* |
| UB19103 | *MAT***a***, ho::LYS2, lys2, ura3, leu2::hisG, his3::hisG, trp1::hisG*  *MATalpha, ho::LYS2, lys2?, leu2::hisG, ura3, his3::hisG, trp1::hisG*  *trp1::TRP1 (C. glabrata)/trp1::TRP1 (C. glabrata)*  *his3::HIS3 (C. glabrata)/his3::HIS3 (C. glabrata)*  *pCUP-IME1::NAT/pCUP-IME1::NAT*  *pCUP-IME4::NAT/pCUP-IME4::NAT* |
| UB19101 | *MAT***a***, ho::LYS2, lys2, ura3, leu2::hisG, his3::hisG, trp1::hisG*  *MATalpha, ho::LYS2, lys2?, leu2::hisG, ura3, his3::hisG, trp1::hisG*  *UME6-IAA7-3V5::KanMX/UME6-IAA7-3V5::KanMX*  *trp1::TRP1 (C. glabrata)/trp1::TRP1 (C. glabrata)*  *his3::HIS3 (C. glabrata)/his3::HIS3 (C. glabrata)*  *pCUP-IME1::NAT/pCUP-IME1::NAT*  *pCUP-IME4::NAT/pCUP-IME4::NAT* |
| UB19105 | *MAT***a***, ho::LYS2, lys2, ura3, leu2::hisG, his3::hisG, trp1::hisG*  *MATalpha, ho::LYS2, lys2?, leu2::hisG, ura3, his3::hisG, trp1::hisG*  *ime1∆::Hyg/ime1∆::Hyg*  *trp1::TRP1 (C. glabrata)/trp1::TRP1 (C. glabrata)*  *his3::HIS3 (C. glabrata)/his3::HIS3 (C. glabrata)* |
| UB22812 | *MAT***a***, ho::LYS2, lys2, ura3, leu2::hisG, his3::hisG, trp1::hisG*  *MATalpha, ho::LYS2, lys2, ura3, leu2::hisG, his3::hisG, trp1::hisG*  *ume6∆::Hyg/ume6∆::Hyg*  *trp1::TRP1 (C. glabrata)/trp1::TRP1 (C. glabrata)*  *his3::HIS3 (C. glabrata)/his3::HIS3 (C. glabrata)* |
| UB21877 | *MAT***a***/MATalpha;  ndt80::pGAL-NDT80::TRP1/ndt80::pGAL-NDT80::TRP1;  ura3::pGPD1-GAL4(848).ER::URA3/ura3::pGPD1-GAL4(848).ER::URA3;*  *ume6::UME6-3V5::HISMX/ume6::UME6-3V5::HISMX;  htb1::HTB1-mCherry::HISMX6/htb1::HTB1-mCherry::HISMX6* |
| UB22674 | *MAT***a***/MATalpha;  ndt80::pGAL-NDT80::TRP1/ndt80::pGAL-NDT80::TRP1;  ura3::pGPD1-GAL4(848).ER::URA3/ura3::pGPD1-GAL4(848).ER::URA3;*  *ume6::UME6-3V5::HISMX/ume6::UME6-3V5::HISMX;*  *cdc20:pCLB2-CDC20::KanMX/cdc20:pCLB2-CDC20::KanMX  htb1::HTB1-mCherry::HISMX6/htb1::HTB1-mCherry::HISMX6* |
| UB11118 | *MAT***a***/MATalpha;  ndt80::pGAL-NDT80::TRP1/ndt80::pGAL-NDT80::TRP1;  ura3::pGPD1-GAL4(848).ER::URA3/ura3::pGPD1-GAL4(848).ER::URA3;  htb1::HTB1-mCherry::HISMX6/htb1::HTB1-mCherry::HISMX6* |

**Table S5. Plasmids used in this study.**

| **Plasmid Name** | **Description** |
| --- | --- |
| pUB1305 | pL245-3V5-IAA17 |
| pUB84 | pFA6a-3V5(SCop)-kanMX6 |
| pUB99 | pNH603-pGPD1 |
| pUB217 | pFA6a-hphNT1 |
| pUB763 | pFA6a-IAA7-3V5-KanMx6 |
| pUB817 | pMJ983 |
| pUB925 | pL399 |
| pUB2441 | 3v5-GFP NanoBody (VH16)–CaURA3 |
| pUB2442 | 8xLexO-osTIR(ScOP)-HIS3 |
| pUB2443 | pIME1-sfGFP-SV40-B112-HIS3 |
| pUB2444 | pIME1-sfGFP-linker-IME1-HIS3 |
| pUB2445 | pIME1-IME1-HIS3 |
| pUB2446 | pIME1-SV40-B112-HIS3 |
| pUB2448 | UME6(T99N)-294-314-gRNA2 |

**Table S6. Oligonucleotides used in this study.**

| **Table S6A: Deletion and C-terminal tagging** | | | |
| --- | --- | --- | --- |
| **Construct name** | **Forward primer** | **Reverse primer** | |
| Ume6 C-terminal Tagging:  Ume6-3v5  Ume6-IAA7-3v5  Ume6^T99N^-3v5  Ume6^T99N^-3v5-aGFP  UB2849 and UB2850: | 5’AAAAACAAAAGAGGCCAAAAGAAGAGCAATGAAAAAAAAA**CGGATCCCCGGGTTAATTAA**3’ | 5’ATAATAATAATAACAATATCTCTTTTTTTTTTTTTCAGTGGAATT**CGAGCTCGTTTAAAC**3’ | |
| *ume6Δ*  UB5676/UB5677: | 5’ACCGCACTCAAACCATTTGCATGGACCTTAACTCACG**CGGATCCCCGGGTTAATTAA**3’ | 5’ATAGTAACAATATCTCTTTTTTTTTTTCAGTGAGCTT**CACTAGTGGATCTGATATCATCG**3’ | |
| *UME6^T99N^* Repair Template  pUB6001/pUB6002 | 5’CTATTATGAAATCGACATGTGCGCCCAACAACAATCCTGTGCATACTCCGTCTGGTTCGC3’ | 5’TTTGGACTTTCAAACTCGGCGAACCAGACGGAGTATGCACAGGATTGTTGTTGGGCGCAC3’ | |
| *UME6^T99N^* gRNA  pUB5999/pUB6000 | 5’AAACTGCATACTCCGTCTGGTTCG3’ | 5’GACTCGAACCAGACGGAGTATGCA3’ | |
| *3V5* Fragment  pUB10419/pUB10420: | 5’AGAACGCGGCCGCCAGCTGAAGCTTCGTACCGGATCCCCGGGTTAATTAA3’ | 5’AGCACCGTCACCGATCCGTCGACCTGCAGCAGCGGTTGAATCTAAACCTA3’ | |
| pUB969 Vector Amplification  pUB10421/pUB3699: | 5’CCCTCCATCCGCGGCCGCTACAAAGCCGAATCCACCACGG3’ | 5’GCCACCGCGGTGGAGCTCTAAGC3’ | |
| *pIME1* Fragment  pUB10422/pUB10423 | 5’TTCGGCTTTGTAGCGGCCGCGGATGGAGGGTTGGCATAAAAATG3’ | 5’CCTGCAGCGTACGCATCCCGGGTTTGTTTGTGGGGAGAGGAATAG3’ | |
| *sfGFP* Fragment  pUB10424/pUB10425: | 5’CTATTCCTCTCCCCACAAACAAACCCGGGATGCGTACGCTGCAGGTC3’ | 5’TCCACCGCGGTGGCTCGAGTCCCTTATAAAGCTCGTCCAT3’ | |
| *SV40-NLS-B112 (sfGFP)* Fragment  pUB10428/pUB10429 | 5’AAGGGACTCGAGATGCCCAAGAAAAAGCGCAAGGTAGAATTTCCAGGTATTACTTTGAG3’ | 5’TATTTGCTTAGAGCTCCACCGCGGTGGTTAAAGCTTGAAACACAAATCAG3’ | |
| *SV40-NLS-B112* Fragment  pUB10430/pUB523 | 5’CCACAAACAAACCCGGGATGCCCAAGAAAAAGCGC3’ | 5’CGCACTCACGTAAACACTTAATC3’ | |
| *sfGFP-IME1* Fragment  pUB10425/pUB10432 | 5’TCCACCGCGGTGGCTCGAGTCCCTTATAAAGCTCGTCCAT3’ | 5’AGAGCTCCACCCTCGAGTTAAGAATAGGTTTTACTAAACTTGTAGGATATTTCTTG3’ | |
| *IME1* Fragment  pUB10431/pUB10432 | 5’CAAACAAACCCGGGATGCAAGCGGATATGCATGG3’ | 5’AGAGCTCCACCCTCGAGTTAAGAATAGGTTTTACTAAACTTGTAGGATATTTCTTG3’ | |
| *OsTIR* Fragment  pUB3870/pUB4098 | 5’TATCAAGCTTATGACTTATTTTCCTGAAGAAG3’ | 5’GAGCTCCACCTCATAAAATCTTGACGAAGTTAGG3’ | |
| *HIS* Vector  pUB10421/pUB4099 | 5’CAAAGCCGAATCCACCACGG3’ | 5’GATTTTATGAGGTGGAGCTCTAAGCAAATAGC3’ | |
| *8x-LexO* Fragment  pUB3915/pUB3869 | 5’CCGTGGTGGATTCGGCTTTGAATAATATATAAACCTGTATAATATAACCTTG3’ | 5’AATAAGTCATAAGCTTGATATCGAATTCC3’ | |
| **Table S6B: qPCR Primers** | | | |
| *UME6 ORF*  pUB7051/pUB7052 | 5’CGCGGTGAAGACCCGTTTGC3’ | | 5’CGGTGGAGGTGGTGGGATGT3’­ |
| *NDT80 ORF*  pUB172/pUB173 | 5’TCTATACAACCGCCCAGCTC3’ | | 5’GACACAAAATGGAGGGCAAT 3’ |
| *IME2 ORF*  pUB415/pUB416 | 5’TGCCTCTTTAGGCGATTCGT3’ | | 5’GCTCGAACTTTTCCCGTGATT3’ |
| *PFY1 ORF*  pUB3301/pUB3302 | 5’ACGGTAGACATGATGCTGAGG3’ | | 5’ACGGTTGGTGGATAATGAGC3’ |
| *pZIP1*  pUB10433/pUB10434 | 5’ACTGCAAGTCTCTGAAAGTTTTAGCTG3’ | | 5’TTCTCTAAAAATTTAGCCGCCGAGG3’ |
| *pSPO13*  pUB10435/pUB10436 | 5’GAGAAATAGCCGCCGACAAAAAGG3’ | | 5’GTGCCATAATTATTCTCGACTCAACTTCAATC3’ |
| *pIME2*  pUB1217/pUB1218 | 5’CCAAATACGCTTTTTAAACTTGG3’ | | 5’CTCAAATAGCCGCCGTAAC3’ |
| *pNUF2*  pUB140 and pUB462 | 5’GAACGCTGATATACTCGACTAAC3’ | | 5’GTCGCTGCGTATTCAGCGTA3’ |
