# Supplementary figures and images for: The transcriptional regulator Ume6 is a major driver of early gene expression during gametogenesis
Source: Genetics. 2023 Jul 11;225(2):iyad123. doi: 10.1093/genetics/iyad123 (PMC10550318; doi:10.1093/genetics/iyad123)

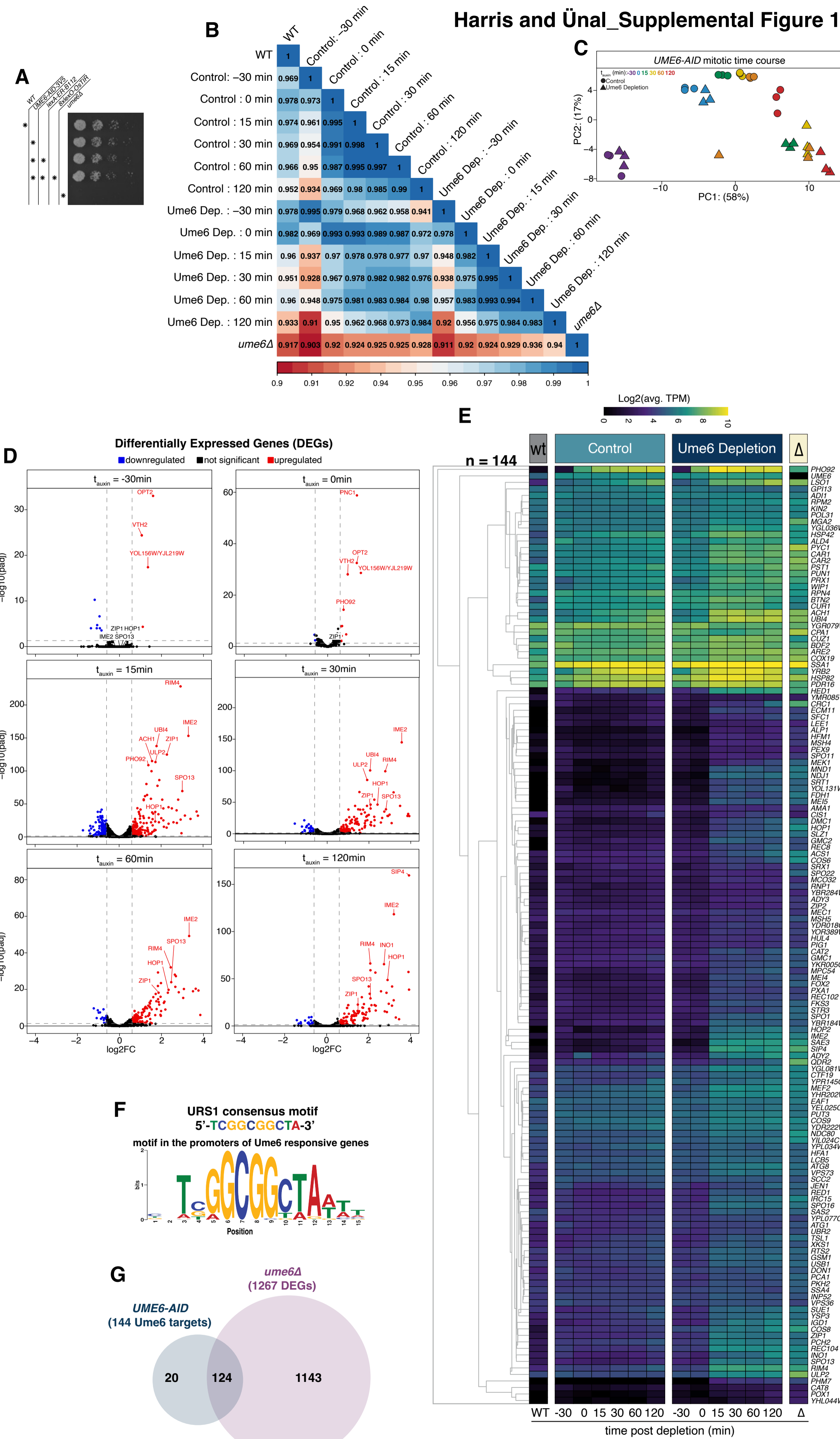

Supplement: iyad123_Supplementary_Data [file iyad123_supplementary_data.zip › Figure_S1_GENETICS-2023-306081.pdf]

# Harris and Ünal\_Supplemental Figure 2

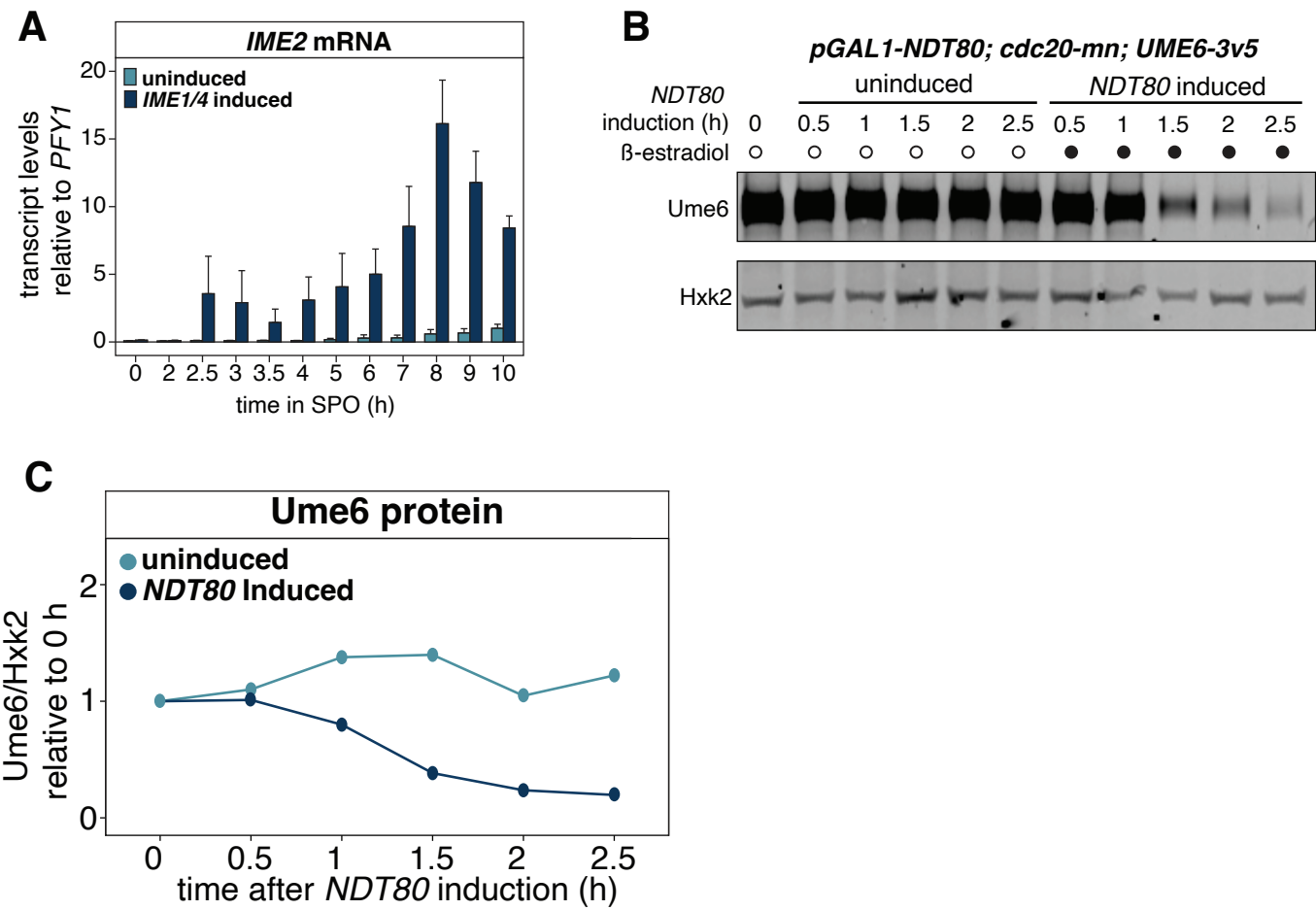

Supplement: iyad123_Supplementary_Data [file iyad123_supplementary_data.zip › Figure_S2_GENETICS-2023-306081.pdf]

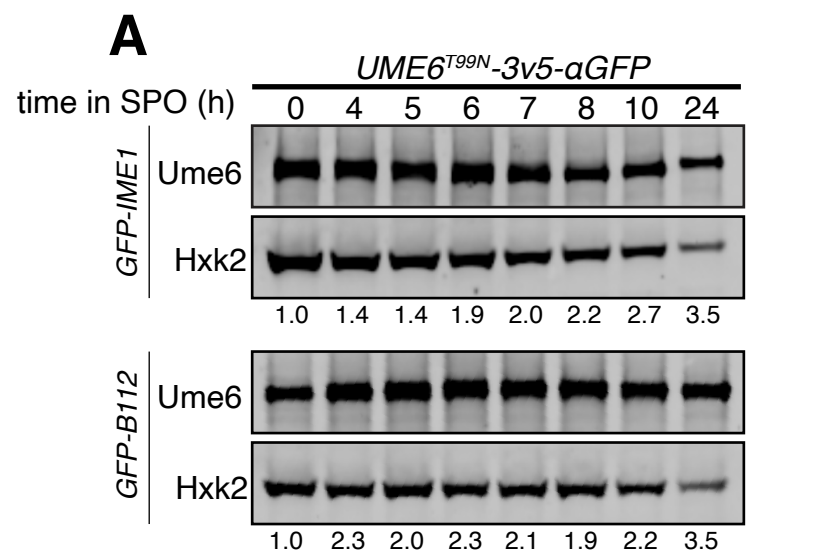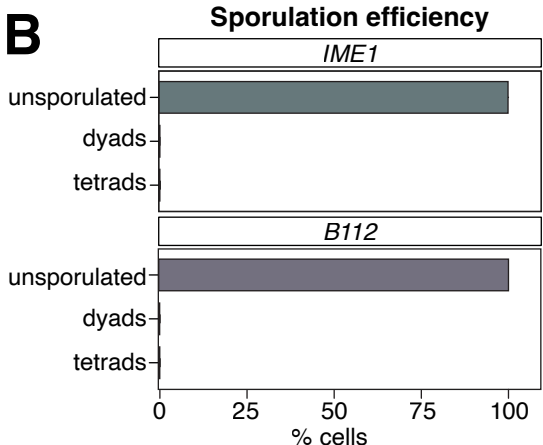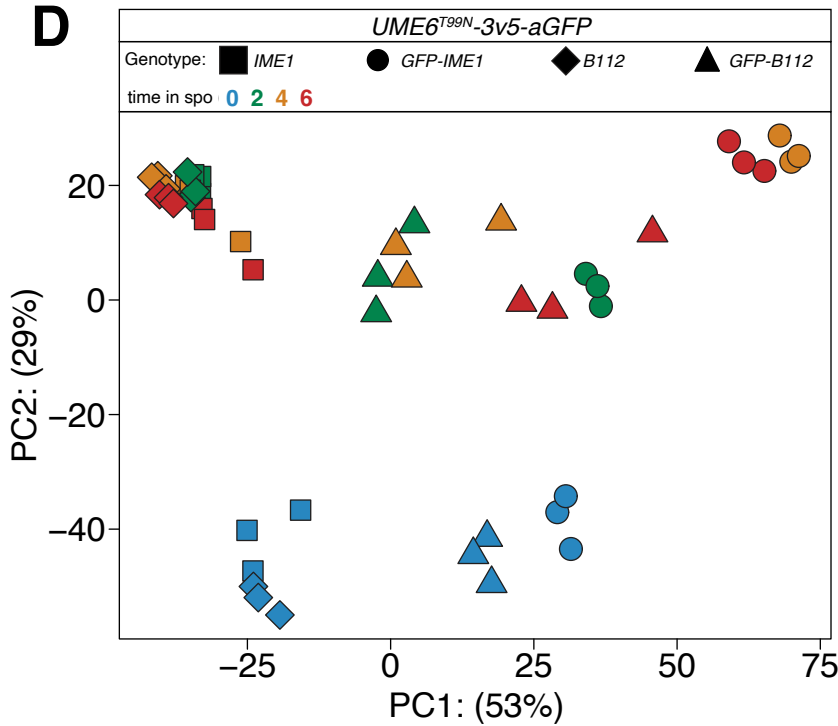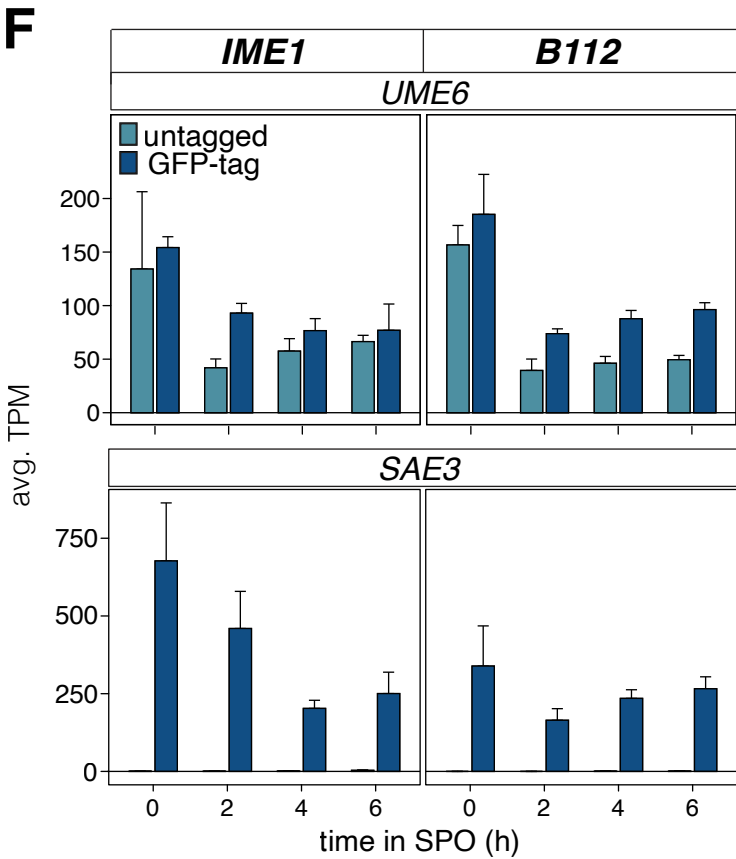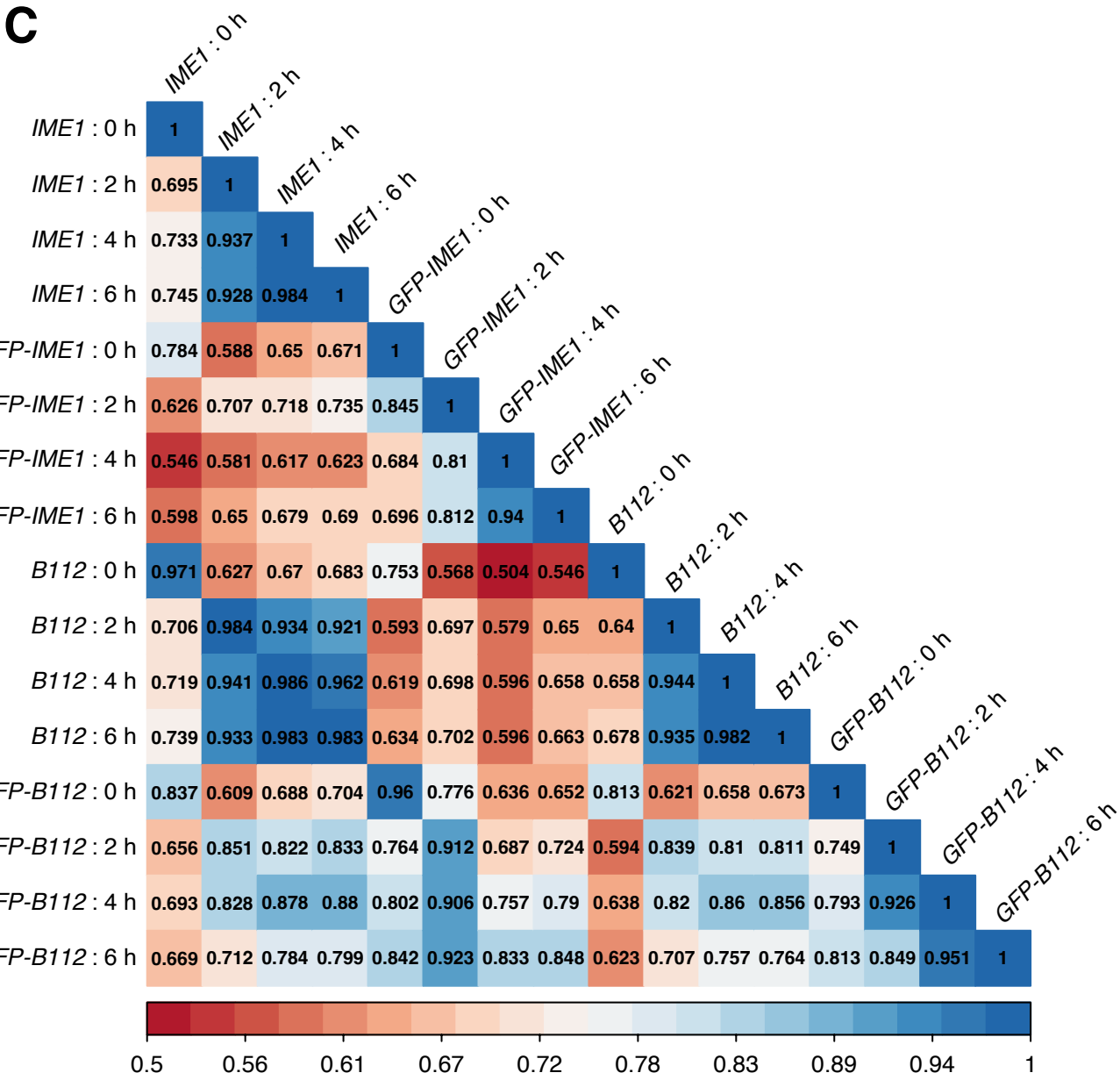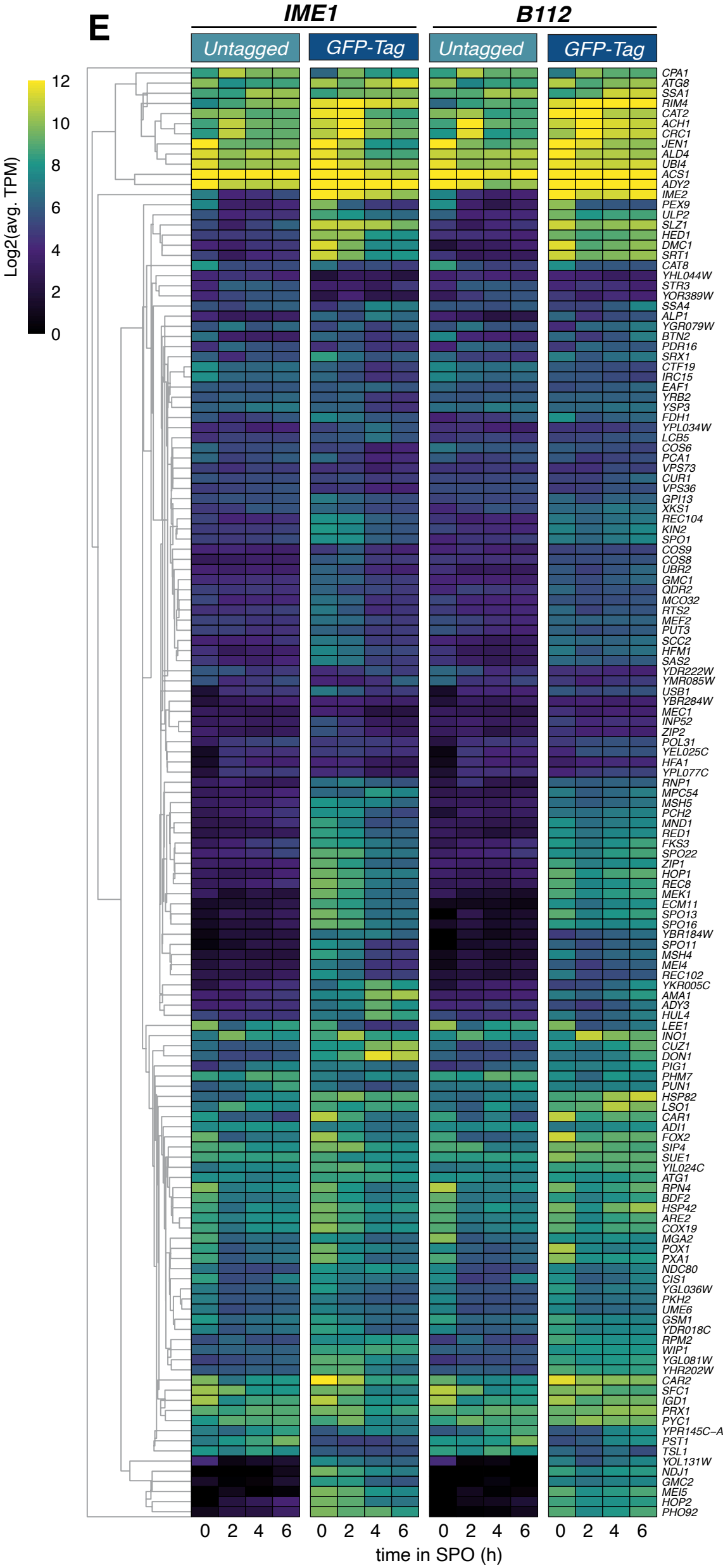

Supplement: iyad123_Supplementary_Data [file iyad123_supplementary_data.zip › Figure_S5_GENETICS-2023-306081.pdf]

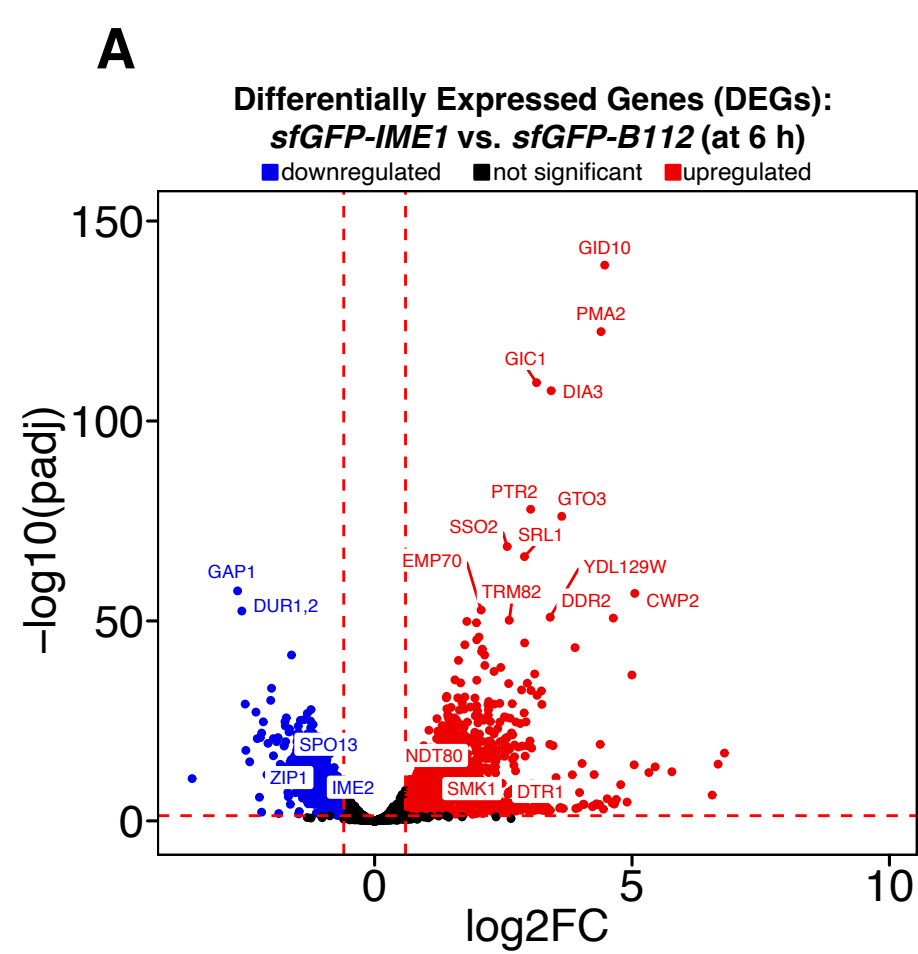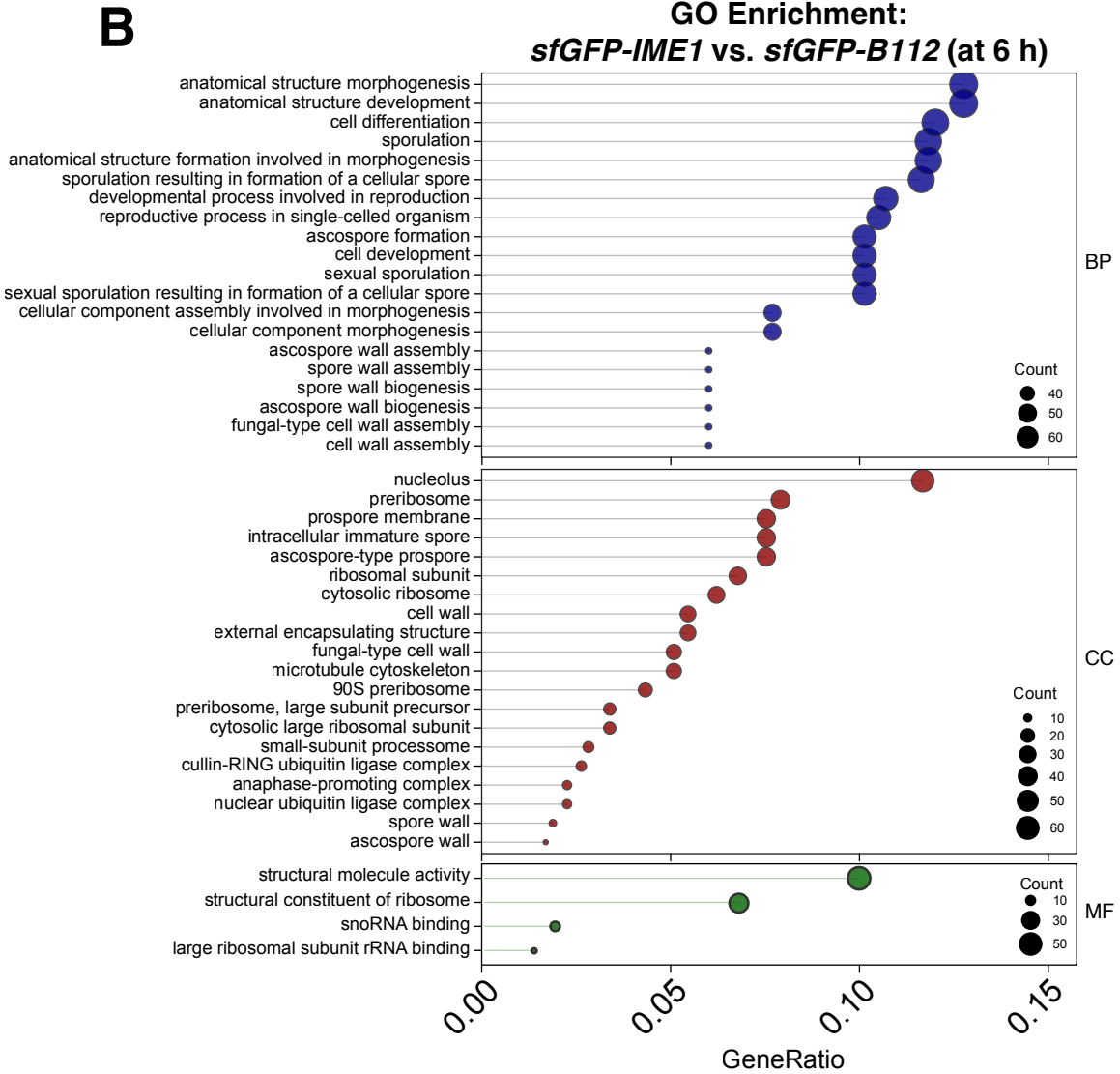

Supplement: iyad123_Supplementary_Data [file iyad123_supplementary_data.zip › Figure_S6_GENETICS-2023-306081.pdf]

# Harris and Ünäl\_Supplemental Figure 4

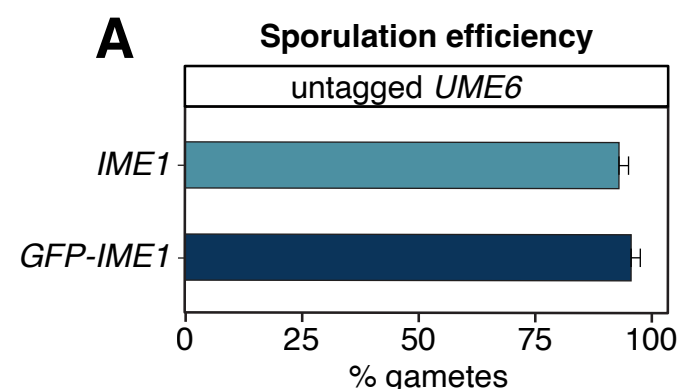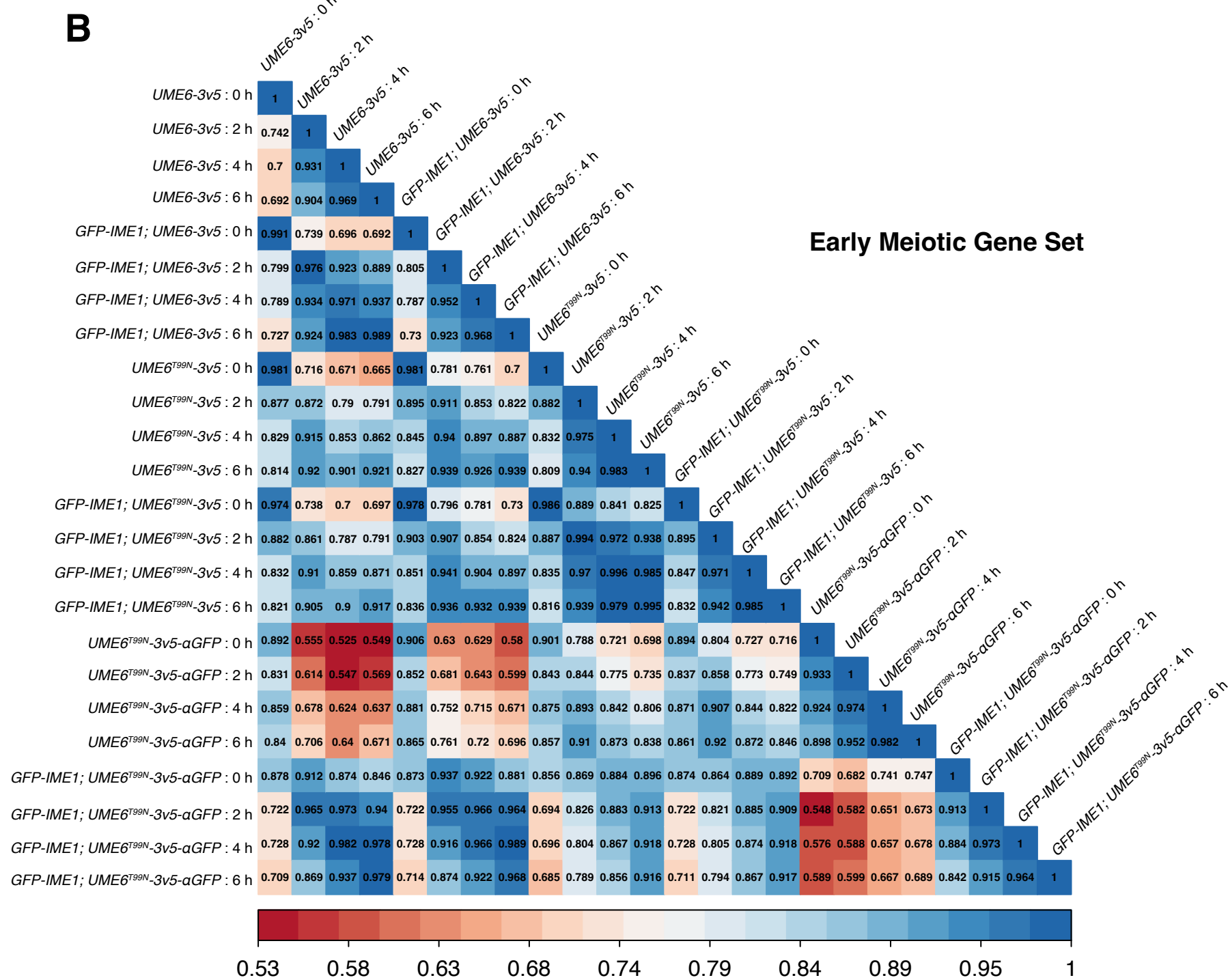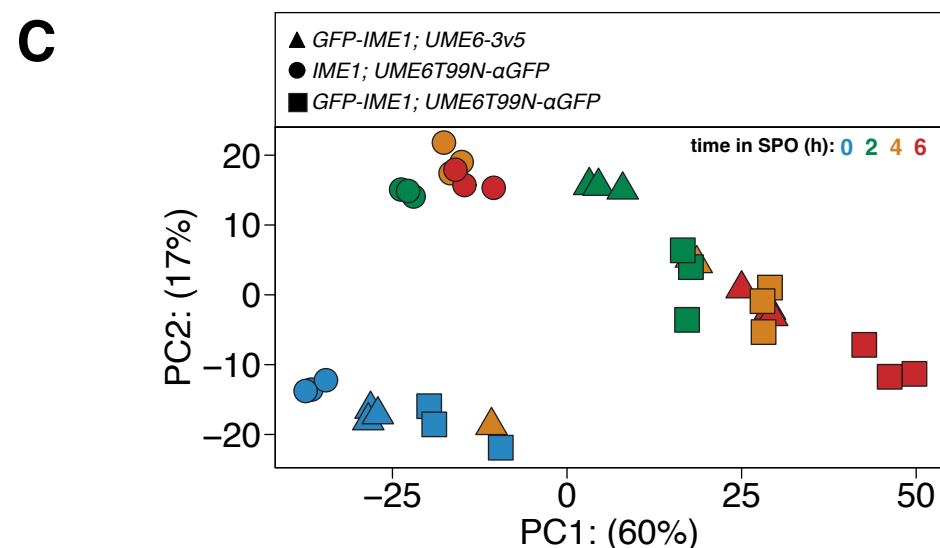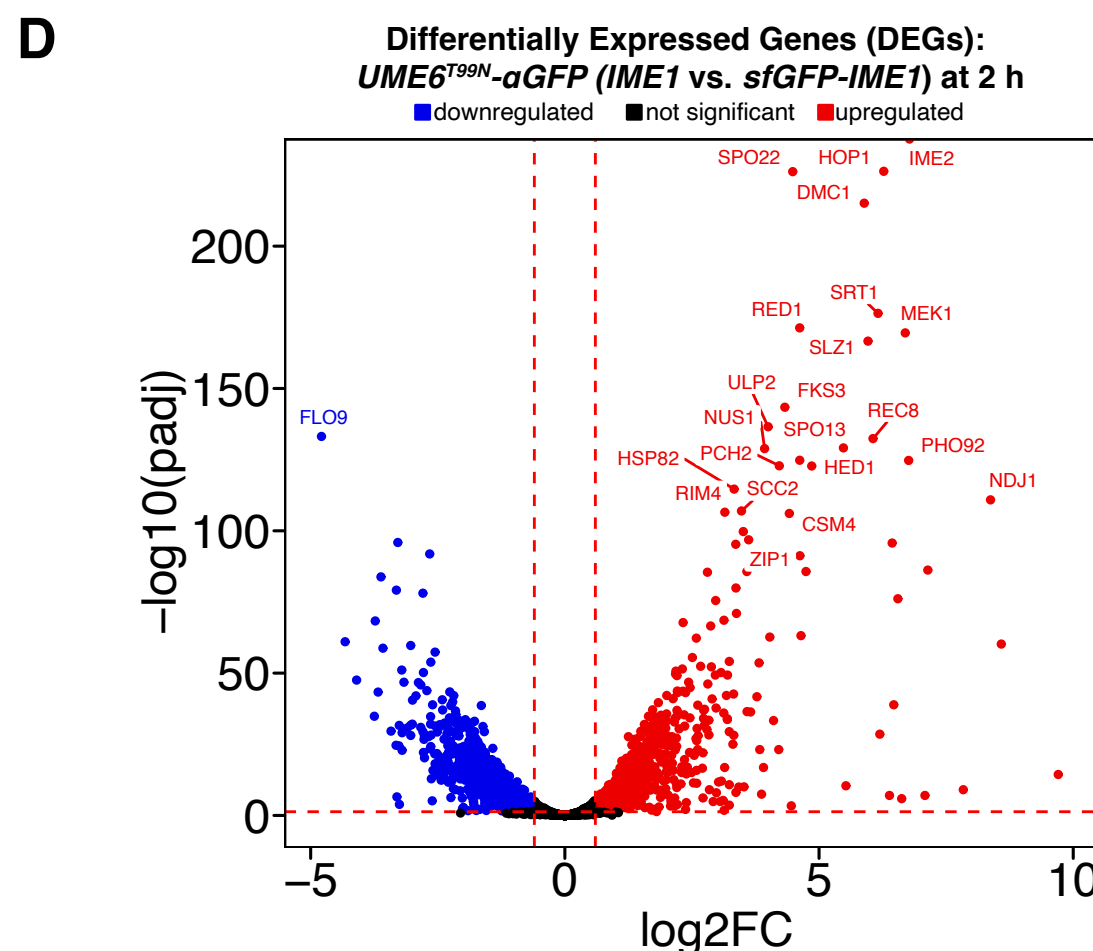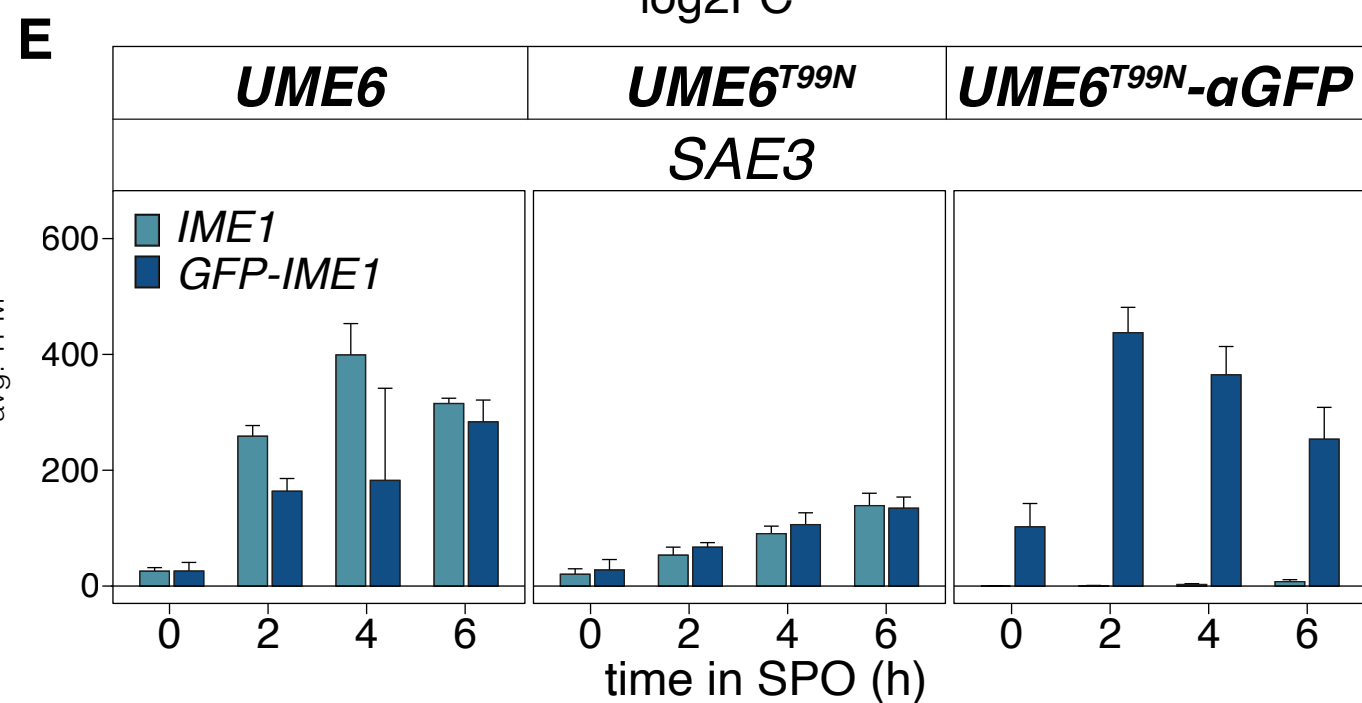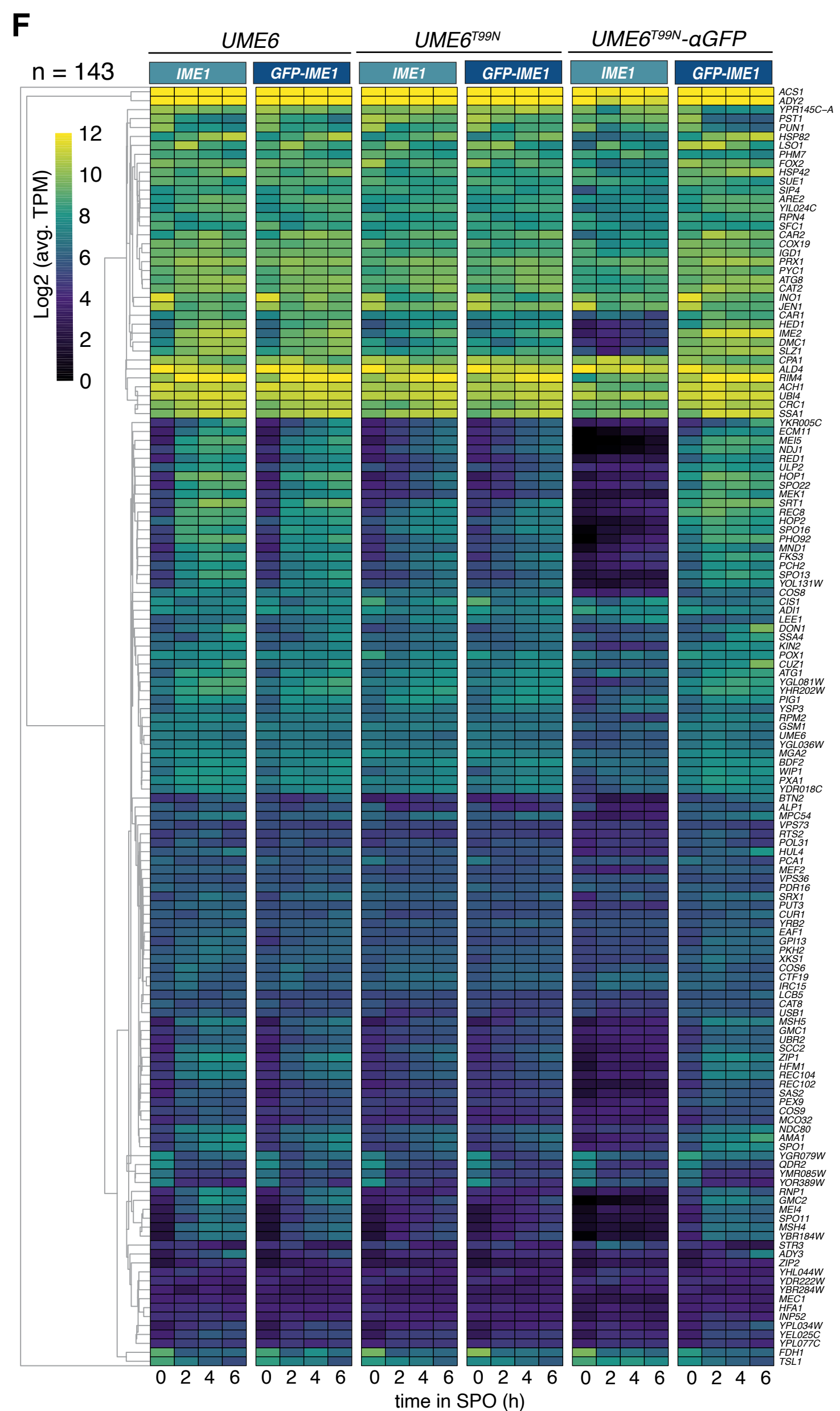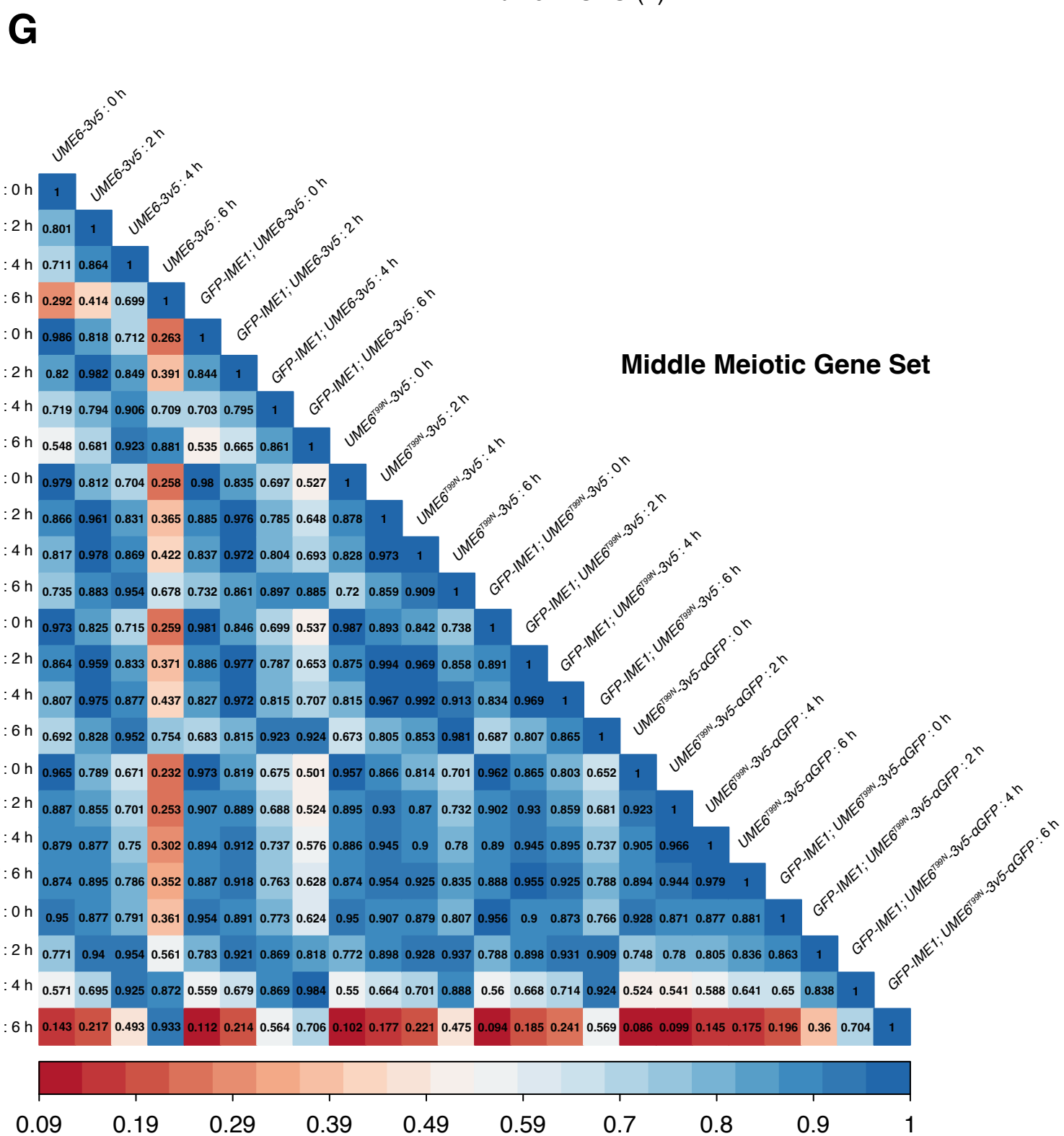

Supplement: iyad123_Supplementary_Data [file iyad123_supplementary_data.zip › HarrisandUnal2023_FigureS4_v2.pdf]
