# Supplementary material for: The transcriptional regulator Ume6 is a major driver of early gene expression during gametogenesis
Source: Genetics. 2023 Jul 11;225(2):iyad123. doi: 10.1093/genetics/iyad123 (PMC10550318; doi:10.1093/genetics/iyad123)

Harris and Ünal_Supplemental Figure 3

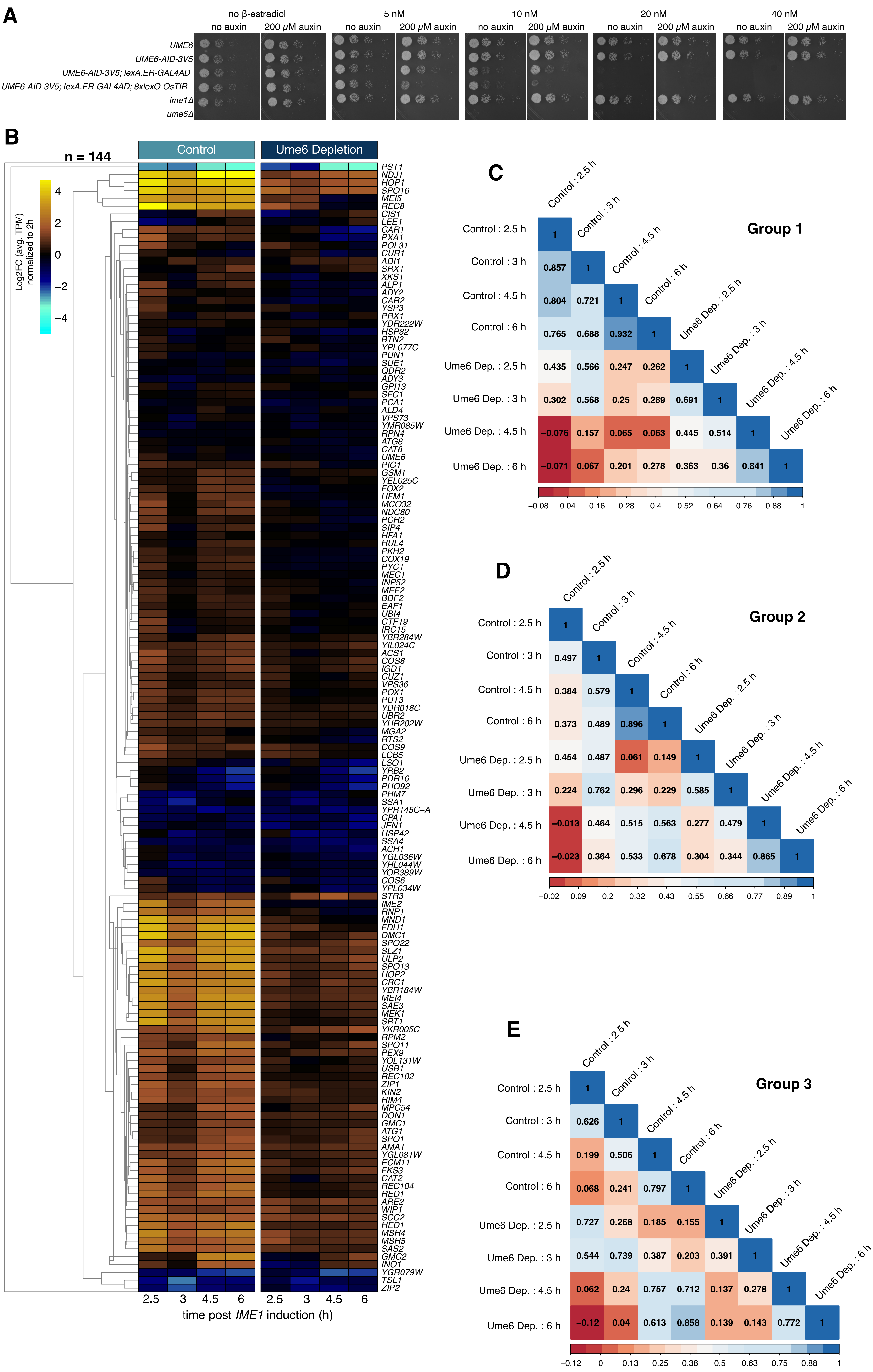

Supplement: iyad123_Supplementary_Data [file Figure_S3_GENETICS-2023-306081.pdf › Figure_S3_GENETICS-2023-306081.pdf]
